# The Emerging Roles of the Calcineurin-Nuclear Factor of Activated T-Lymphocytes Pathway in Nervous System Functions and Diseases

**DOI:** 10.1155/2016/5081021

**Published:** 2016-08-15

**Authors:** Maulilio John Kipanyula, Wahabu Hamisi Kimaro, Paul F. Seke Etet

**Affiliations:** ^1^Department of Veterinary Anatomy, Faculty of Veterinary Medicine, Sokoine University of Agriculture, P.O. Box 3016, Chuo Kikuu, Morogoro, Tanzania; ^2^Department of Basic Health Sciences, Qassim University, Buraydah, Al-Qassim 51452, Saudi Arabia

## Abstract

The ongoing epidemics of metabolic diseases and increase in the older population have increased the incidences of neurodegenerative diseases. Evidence from murine and cell line models has implicated calcineurin-nuclear factor of activated T-lymphocytes (NFAT) signaling pathway, a Ca^2+^/calmodulin-dependent major proinflammatory pathway, in the pathogenesis of these diseases. Neurotoxins such as amyloid-*β*, tau protein, and *α*-synuclein trigger abnormal calcineurin/NFAT signaling activities. Additionally increased activities of endogenous regulators of calcineurin like plasma membrane Ca^2+^-ATPase (PMCA) and regulator of calcineurin 1 (RCAN1) also cause neuronal and glial loss and related functional alterations, in neurodegenerative diseases, psychotic disorders, epilepsy, and traumatic brain and spinal cord injuries. Treatment with calcineurin/NFAT inhibitors induces some degree of neuroprotection and decreased reactive gliosis in the central and peripheral nervous system. In this paper, we summarize and discuss the current understanding of the roles of calcineurin/NFAT signaling in physiology and pathologies of the adult and developing nervous system, with an emphasis on recent reports and cutting-edge findings. Calcineurin/NFAT signaling is known for its critical roles in the developing and adult nervous system. Its role in physiological and pathological processes is still controversial. However, available data suggest that its beneficial and detrimental effects are context-dependent. In view of recent reports calcineurin/NFAT signaling is likely to serve as a potential therapeutic target for neurodegenerative diseases and conditions. This review further highlights the need to characterize better all factors determining the outcome of calcineurin/NFAT signaling in diseases and the downstream targets mediating the beneficial and detrimental effects.

## 1. Introduction

Cellular responses to calcium (Ca^2+^) mobilization are highly versatile due to the ability of intracellular Ca^2+^ signaling to activate an extensive repertoire of downstream signaling targets [1, 2]. Among such molecules, the calmodulin- (CaM-) dependent phosphatase calcineurin and its transcription factors termed nuclear factors of activated T cells (NFATs) are reported as pivotal in a wide range of physiological processes, including homeostasis, angiogenesis, myogenesis, adipogenesis, osteogenesis, chondrocyte differentiation, cardiovascular system development, pancreatic *β*-cell proliferation, hair follicle cell differentiation and remodeling, and activities of cells of the immune and nervous systems [3–12]. Overexpressed and decreased activities of calcineurin/NFAT pathway were also reported in pathologies affecting these functions [3, 6, 13–18].

A growing body of evidence suggests that calcineurin/NFAT pathway plays critical roles in normal and pathological nervous system. Over the last decade, this signaling pathway was reported as major player in corticogenesis, synaptogenesis, and neuritogenesis during mammalian nervous system development [19–23], as well as myelination, synaptic plasticity, neurotransmission, and central and peripheral nervous system cell proliferation, migration, and differentiation in the mature nervous system [24–26]. Altered calcineurin/NFAT activation is increasingly linked to pathological features of neurodegenerative diseases such as amyotrophic lateral sclerosis, Huntington's, Parkinson's, and Alzheimer's diseases, characterized by massive synaptic dysfunction, glial activation, and neuronal death in some regions of the brain [26–29]. Calcineurin/NFAT involvement has also been reported in psychiatric disorders, epilepsy, and traumatic brain and spinal cord injuries [30–35]. Moreover, recent data also suggest that NFAT isoforms are selectively activated in neurons and glial cells in nervous system diseases [36, 37] and animal models [38–41].

Herein, we discuss the current understanding of the role of calcineurin/NFAT signaling pathway in both physiology and pathologies of the adult and developing nervous system, with an emphasis on recent reports and cutting-edge findings.

## 2. Calcineurin/NFAT Pathway

### 2.1. Calcineurin

Calcineurin, also termed as protein phosphatase B (PP2B), is a Ca^2+^/CaM-dependent serine/threonine phosphatase. It was described for the first time in the bovine brain about 40 years ago [42, 43]. Calcineurin is made up of a 61 kD CaM-binding catalytic subunit termed calcineurin A (CnA) and a 19 kD Ca^2+^-binding regulatory subunit named calcineurin B (CnB) stably associated (noncovalently) [44, 45]. To date three isozymes of the catalytic subunit (CnA*α*, CnA*β*, and CnA*γ*) have been reported. There are two isoforms of the regulatory subunit, namely, CnB1 and CnB2. In vertebrates, each subunit is encoded by a separate gene PPP3CA, PPP3CB, and PPP3CC for CnB1 and PPP3R1, PPP3R2 for CnB2 [46, 47]. The two calcineurin isoforms are widely distributed in mammalian tissues. Immunohistochemical studies showed that under normal conditions calcineurin isoforms are highly expressed in neuroinflammation-sensible neurons like corticohypothalamic pyramidal cells and cerebellar Purkinje cells, as well as in peripheral nervous system (PNS) glia like Schwann cells, but not in central nervous system (CNS) glia [37, 48, 49].

The subcellular distribution of calcineurin is an important control point in regulating its activity. Various studies addressed the subcellular localization of calcineurin in various mammalian cells [2, 50, 51]. In neurons, calcineurin was found in the cytoplasm, endoplasmic reticulum, Golgi apparatus, nucleus, synaptic vesicles, microsomes, mitochondrion outer membrane, and plasma membrane [2, 48, 49, 51, 52].

### 2.2. NFAT Transcription Factors

NFAT proteins are a family of transcription factors normally found in the cytoplasm in a hyperphosphorylated (inactive) state [53–55]. This family comprises five distinct gene products termed (i) NFATc, NFAT2, or NFATc1; (ii) NFATp, NFAT1, or NFATc2; (iii) NFATx, NFAT4, or NFATc3; (iii) NFAT3 or NFATc4; and (iv) osmotic response element-binding protein (OREBP), tonicity-responsive binding-protein (TonEBP), or NFAT5 [56, 57]. In the context of this review, nomenclature of NFATc1-c4 and NFAT5 will be used.

NFATc1–c4 occur as monomers with unique amino and carboxyl termini containing transcription activation domains (TADs) and two conserved domains: (i) a regulatory domain termed NFAT homology region (NHR), which shows a lesser degree of pair-wise sequence identity but has several strongly conserved sequence motifs characteristic of the NFAT family; and (ii) the Rel homology region (RHR) in the C-terminus, where the DNA binding domain (DBD) is located [14, 56]. The NHR encompasses calcineurin docking sites, a nuclear localization signal (NLS) responsible for calcineurin-mediated nuclear translocation, and an extended serine-rich region [44, 58–61]. On the other hand, the DBD binds DNA and interacts with partner proteins to transactivate gene transcription. Examples of partner transcription factors include AP-1 cell life and death regulators [dimeric transcription factors composed of activating transcription factor (ATF), Fos, or Jun subunits] and important oncogenic regulators like myocyte enhancer factor-2 (MEF2) and GATA binding protein 4 (GATA4) [60, 62]. Regions out of the regulatory and DNA binding domains like TADs demonstrate relatively little sequence conservation [57, 59]. NFAT5 is a homodimer with a distinct domain structure. This isoform retains only the RHR region of homology to the Ca^2+^-regulated isoforms, whilst the remaining 600 amino acids are completely different. The DBD of NFATs is distantly related to the DBD of nuclear factor kappa B (NF-*κ*B)/Rel family, allowing them to be classified sometimes as members of this extended family [63, 64].

NFATs regulate the transcriptional induction of genes encoding for immune modulators/activators such as granulocyte-macrophage colony-stimulating factor (GM-CSF), forkhead box P3 (FOXP3), immunoglobulin kappa (Ig*κ*), gamma interferon (IFN*γ*), CD5, CD25, CD28, CD40, interleukin- (IL-) 2, IL-3, IL-4, IL-5, IL-13, IL-8, Th2-type cytokine IL-31, Fas ligand, macrophage inflammatory protein 1 alpha (MIP-1*α*), protein tyrosine kinase Syk, cyclooxygenase 2 (COX-2), and tumor necrosis factor alpha (TNF-*α*) and its family member BlyS. NFATs may also control genes encoding signaling molecules as variate as Ca^2+^ regulators [inositol 1,4,5-trisphosphate (IP_3_) receptor (IP_3_R), regulator of calcineurin 1 (RCAN1)], growth factors (VEGF, neurotrophins), myelination genes (P0 and Krox-20), glucose regulation genes (insulin, HNF1, PDX, and GLUT2), cell cycle and death regulator/activators [p21^Waf1^, c-Myc, cyclin-dependent kinase 4 (CDK4), B-cell lymphoma 2 (Bcl-2), and cyclins A2, D1, and D2], oncogenes (Wnt, *β*-catenin), microRNAs (miR-21, miR-23, miR-24, miR-27, miR-125, miR-195, miR-199, and miR-224), and surfactants (sftpa, sftpb, sftpc, and abca3) [9, 65–74]. NFAT isoforms are ubiquitously expressed and are generally regulated by Ca^2+^ signaling, with exception of NFAT5 [59, 75, 76]. NFAT5 regulates hypertonic stress-induced gene transcription, whereas the other NFATs act as integrators of Ca^2+^ driven signaling pathways in gene expression and cell differentiation programs [57, 77, 78].

All Ca^2+^-regulated NFATs (NFATc1–c4) are expressed in neurons [79]. The previously assumed functional redundancy of NFAT functions was proved wrong by their wide-ranging expression profile among cell types under both physiological and pathological conditions. For example, loss of specific NFAT subtypes resulted in cardiovascular, skeletal muscle, cartilage, neuronal, and/or immune system defects [54, 59, 80, 81]. In addition, Vihma and colleagues reported that NFAT subcellular localizations and transcriptional activities are isoform- and cell type-specific [37]. In that context, the strongest transcriptional activators were NFATc3 and NFATc4 in primary hippocampal neurons and NFATc1 and NFATc3 in human embryonic kidney-derived HEK293 cells.

### 2.3. Signaling Activation

The activation of calcineurin/NFAT signaling pathway involves three key steps: (i) NFAT protein dephosphorylation by calcineurin; (ii) nuclear translocation of NFATs; and (iii) increased affinity for DNA. Ligand-receptor interaction activates phospholipase C (PLC) resulting in the release of IP_3_, which in turn leads to the release of Ca^2+^ from intracellular stores through IP_3_Rs [59, 82]. Notably, Ca^2+^ release from the intracellular stores requires a stimulus capable of generation of second messengers that trigger Ca^2+^ release from intracellular stores in the endoplasmic/sarcoplasmic reticulum, via channels of IP_3_R1–3 and ryanodine receptors (RyR1–3) [59, 83–87]. Like for other Ca^2+^/CaM-dependent enzymes, calcineurin activation requires increase in cytosolic Ca^2+^ levels. Such activation may result from the binding of Ca^2+^ to calcineurin B subunit or from the binding of Ca^2+^-activated CaM to calcineurin [50]. In turn, calcineurin activates NFATs by dephosphorylating multiple N-terminal phosphoserine residues in the regulatory domain. Such dephosphorylation increases NFAT affinity for DNA [59]. The dephosphorylation results in a conformational change in the NFAT molecule that exposes the nuclear localization signal, allowing NFAT nuclear translocation to take place, presumably through nuclear pores [88]. In the nucleus NFATs become transcriptionally active by forming complexes with other factors and coactivators, providing a direct link between intracellular Ca^2+^ signaling and gene expression. Calcineurin also promotes nuclear retention of NFAT by masking nuclear export signals (NES) and preventing NES-dependent nuclear export [88, 89]. Moreover, calcineurin mediated NFAT activation is mainly required at resting state (resting membrane potential), where the amount of Ca^2+^ released from intracellular stores is not sufficient for direct NFAT activation [75, 90].

### 2.4. Regulation and Pharmacology

#### 2.4.1. Endogenous Regulation

Regulators of calcineurin (RCANs) or modulatory calcineurin-interacting proteins (MCIPs; MCIP1–3) belonging to the calcipressin family of proteins play a pivotal role in regulating calcineurin activity. The RCANs are evolutionarily conserved proteins that can directly bind and inhibit calcineurin. The RCAN1 genes are found on chromosome 21. The RCAN gene encodes different isoforms of protein, namely, RCAN1, RCAN2, RCAN3, and RCAN4 where RCAN1 and RCAN4 are the main isoforms. Furthermore, it appears that RCAN1 has two RCAN1 isoforms: 1S with 197 amino acids and RCAN.1L with 252 amino acids. Previous studies have shown that an aberration in RCANs activities decreases calcineurin-NFAT signaling activities. Recent studies have shown that RCANs can show both inhibitory and facilitatory roles in activation of the calcineurin-NFAT signaling pathway. A mechanistic explanation on how RCAN proteins precisely modulate calcineurin function is still debatable. Overall RCANs have been implicated to function primarily as chaperones for calcineurin biosynthesis or recycling, requiring binding, phosphorylation, ubiquitylation, and proteasomal degradation for their stimulatory effect [50, 91, 92]. Other regulators including scaffolding proteins, CAIN/CABIN-1, and A-kinase anchoring protein 79 (AKAP79) have also been identified to interact and inhibit calcineurin function in a phosphorylation-dependent manner in mammalian cells (details given below).

#### 2.4.2. Endogenous Inhibition

The regulation of calcineurin/NFAT activity is achieved: (i) in the nucleus by activities of serine/threonine kinases promoting the export of nuclear NFAT; (ii) in the cytoplasm, through phosphorylation of NFAT serine (SP) repeats and N-terminal domain, which are critical for NFAT activation and nuclear import [88, 93, 94]. Various kinases were implicated in NFAT nuclear export. Examples include glycogen synthase kinase (GSK), protein kinase A (PKA), casein kinase, and also mitogen-activated protein kinases (MAPKs) like c-Jun NH_2_- terminal kinases (JNKs) and cellular stress-associated p38 kinase [14, 54]. Although different kinases have been implicated in regulating NFAT activity, the distinction between drivers and passengers in the cytoplasm and in the nucleus is still puzzling. Notably, it is not clearly understood whether the kinases which mediate rephosphorylation of nuclear or activated NFATs are similar to those that phosphorylate NFATs under basal state conditions [14, 54]. Other early reports from targeted genetic and pharmacological manipulations suggest that members of the JNK family regulate both the import and export of NFATs while p38 MAPKs mediate NFAT rephosphorylation, thus NFAT nuclear export [13, 89, 95].

Calcineurin/NFAT signaling blocking agents mainly act at calcineurin and NFAT levels. They include cellular (protein inhibitors) and pharmacological inhibitors. Although numerous endogenous proteins may have potential to inhibit calcineurin or NFAT activities, only four are well-characterized: (i) A-kinase anchoring protein 79 (AKAP79), a scaffold protein that prevents calcineurin-substrate interactions [96, 97]; (ii) calcineurin inhibitor (CAIN) or calcineurin-binding (CABIN) proteins, which block calcineurin activity [98, 99]; (iii) calcineurin homologous protein (CHP); and (vi) modulatory calcineurin-interacting proteins (MCIP1–3), which prevent NFAT nuclear import by preventing its phosphorylation [94, 100–103].

#### 2.4.3. Pharmacological Inhibition

Two pharmacological inhibitors of NFAT translocation, namely, cyclosporine A, FK506 (tacrolimus), and its ethyl analog ascomycin, are commonly used as immunosuppressants, particularly in organ transplantation [104–107]. These chemically distinct microbial products inhibit calcineurin activity by binding with subnanomolar affinity to cytosolic proteins called immunophilins. The resultant drug-protein composite binds tightly to calcineurin and blocks its phosphatase activity by preventing substrate access [14, 59]. Cyclosporine A binds to cyclophilin and FK506 binds to FK506-binding protein (FKBP) [88, 108]. These inhibitors indiscriminately block all downstream calcineurin signaling, including various signaling pathways collaborating with calcineurin/NFAT signaling pathway [39, 93]. Such complete blockade of calcineurin activities may explain at least partly the serious side effects of cyclosporine A and FK506 [104, 105], particularly in pediatric patients [106, 107].

Recent studies in rat brain slices and cultured astrocytes revealed that cyclosporine A increases reactive oxygen species (ROS) formation and alters glucose and energy metabolism partly by Krebs cycle inhibition and anaerobic glycolysis activation [109]. These detrimental effects probably participate in cyclosporine A neurotoxicity. Additionally, inhibition of NFAT activity suppressor GSK-3 mediated by wild-type mice chronic treatment with lithium or cyclosporine A resulted in increases in nuclear translocation of NFATc3 and Fas-dependent apoptosis in brain neurons, accompanied by pronounced motor deficits [110]. In the same study, neither neuronal loss nor motor deficits were observed in Fas deficient Tet/DN-GSK-3 mice, suggesting that GSK-3 contributes to the neurological toxicity induced by cyclosporine A. Therefore, GSK-3 inhibitors may improve calcineurin inhibitor neurotoxicity. Cyclosporine A may also mediate neuronal affection by decreasing biometal availability [111–113].

Moreover, experimental evidence has shown increases in spinal NMDA receptor activity as a result of calcineurin inhibitor induced pain syndrome (CIPS) [114–116]. A whole-cell patch-clamp study in spinal cord slices revealed that the effect may be mediated by the potentiation of pre- and postsynaptic NMDA receptor activity in the spinal cord [114]. In the same study, it was shown that FK506 treatment increased drastically the amplitude of excitatory postsynaptic currents mediated by NMDA receptor in dorsal horn neurons. Inhibitors of the serine/threonine protein kinase casein kinase II (CK2) involved in the upregulation of synaptic NMDAR activity in neurons abrogated pain hypersensitivity caused by FK506 [117, 118]. Nonetheless, cyclosporine A also mediated neuropathic pain independently of NMDA receptor [108, 118], suggesting that the processes mediating CIPS are complex and warrant further studies.

Interestingly, inhibitors that do not block calcineurin enzymatic activity* per se*, but rather interfere with enzyme targeting of one or more of its substrates, have recently been developed and used. For instance, the inhibitors of NFAT-calcineurin association (INCA) compounds may interfere selectively with calcineurin/NFAT interaction without preventing dephosphorylation of other calcineurin substrates [119, 120]. Substrate-selective enzyme inhibition represents an important progress over cyclosporine A or FK506-mediated complete blockade of calcineurin/NFAT signaling. This development is expected to lead to the development of safer classes of calcineurin/NFAT inhibitors. Certainly, the beneficial actions of cyclosporin A and FK506 are counterbalanced by serious toxicities attributed partly to their interference with calcineurin signaling in other cells and tissues. Although INCA have nonspecific cytotoxic effects, they are generally considered to be less toxic than treatment with cyclosporine A or FK506.

## 3. Nervous System Development

Calcineurin/NFAT signaling pathway is multifunctional. In the PNS, calcineurin/NFAT signaling was reported to have critical roles in the survival, proliferation, and differentiation of both neural and glial precursor cells [22], highlighting its potential role in tissue regeneration. Similar lines of evidence suggest that calcineurin/NFAT plays critical roles in the regulation of the CNS development, including corticogenesis and synaptogenesis.

### 3.1. Corticogenesis

Evidence supporting the involvement of the calcineurin/NFAT4 signaling pathway in corticogenesis includes a study in developing mouse cerebellar granule neurons that reported a pivotal role in controlling the temporal regulation of nuclear factor 1 occupancy. This serves as a key link between membrane potential and dendritic maturation, by a voltage-sensitive developmental switch [121]. In addition, the multifunctional HMG-box transcription factor Tox, a novel regulator of mammalian corticogenesis, is regulated by calcineurin/NFAT signaling [19]. Genetic and biochemical analyses in the developing embryo revealed that fibroblast growth factor- (FGF-) mediated calcineurin signaling may trigger neural induction by increasing Smad1/5 transcription via silencing of bone morphogenetic protein (BMP) signaling [122]. Furthermore, store-operated Ca^2+^ entry (SOCE) activation regulates gene transcription in the developing nervous system and mediates neural progenitor cell proliferation through calcineurin/NFAT signaling [123–125].

The role of the calcineurin/NFAT signaling in corticogenesis is probably mediated by NFATc3, the predominant NFAT isoform in neural progenitor cell cultures, which is also a potent inducer of neural progenitor cell differentiation into neurons and astrocytes [22, 126]. Interestingly, activity-dependent NFATc3 accumulation in the nucleus was reported in pericytes from cortical parenchymal microvessels [127], and differential expressions of NFATc3 and NFATc4 were reported in developing rat brain and traumatic brain injury models, where NFATc4 was primarily expressed by neurons and NFATc3 by astrocytes [125, 128]. These observations suggest that different NFATs are recruited at the same time in resident cells of damaged and growing nervous tissue, indicating that characterizing the effects of such NFAT changes in specific cell types may provide new therapeutic targets for neurodevelopmental disorders.

### 3.2. Synaptogenesis

The complex interactions between inhibitory gamma-aminobutyric acid (GABA) and excitatory NMDA receptor activities are required during synaptogenesis. Furthermore, this interaction plays an important role in the induction of immediate early genes necessary for effective changes in synaptic plasticity and long-term memory formation through calcineurin-dependent transcription of the key brain-derived neurotrophic factor (BDNF) [20, 23, 129, 130]. Of particular interest for neurodevelopmental treatments is a recently reported novel synthetic neurotrophic (BDNF and neurotrophin-like) compound that is able to induce neurite growth and confer neuroprotection [21]. In addition, the neurotrophin nerve growth factor (NGF) is known to upregulate the key regulator of plasminogen activation system and synaptogenic protein plasminogen activator inhibitor 1 (PAI-1) in primary mouse hippocampal neurons via calcineurin/NFAT signaling [131]. Protein kinase C (PKC)/calcineurin signaling-mediated dephosphorylation of axon growth regulatory molecule growth-associated protein 43 (GAP43) at developing GABAergic synapses resulted in pathological processing mimicking neonatal hypoxia, including misfolding of gephyrin, a protein critical for the organization of GABA receptors [132]. Calcineurin signaling also mediates the GABAergic synaptic modulation induced by transient receptor potential vanilloid type 1 (TrpV1), a ligand-gated channel abundantly expressed in developing primary sensory neurons [133, 134]. Certainly, these observations suggest that calcineurin signaling is involved in the development of GABAergic synaptic functions in the CNS.

Moreover, GABA promoted the shrinkage and elimination of synapses by suppressing local dendritic Ca^2+^ signaling in rat hippocampal CA1 pyramidal neurons via a mechanism depending on calcineurin and on actin-binding protein cofilin [135]. Similarly, more recent studies also suggested that calcineurin is a major signaling molecule in the selection of synapses, a critical mechanism in the reorganization of the developing and adult CNS mediated by the major inhibitory neurotransmitter GABA [132, 135, 136].

### 3.3. Endosome Trafficking

It is now widely accepted that calcineurin signaling is a major player in the control of the trafficking and signaling of endosomes performing the retrograde signaling, an event critical for the development, but also for the nervous system function [2, 137, 138]. Calabrese and colleagues reported calcineurin signaling modulation as a key event in the differential regulation of dynamins, major players of synaptic vesicle recycling in nerve terminals of developing neurons [137]. Notably, these authors observed that tetrodotoxin- (TTX-) mediated chronic suppression of neuronal activity results in the suppression of dynamin 1 clustering at nerve terminals and in an increase of clustering of dynamin 3, partly mediated by calcineurin signaling silencing. In addition, NGF-mediated calcineurin/NFAT signaling is critical for the control of endosome trafficking in neurons [77, 131, 138]. Such NGF/calcineurin/NFAT control of the trafficking of endosomes is under the control of the effector protein coronin-1 and regulatory events such as the phosphorylation of cAMP responsive element binding protein (CREB), which are also mediated by NGF receptor tropomyosin receptor kinase type 1 (TrkA) [138].

Notably, as other tyrosine kinase receptors, the receptors of neurotrophins may also activate NFATs via inhibition of the promoter of NFAT nuclear export GSK-3*β*, independent of Ca^2+^ and calcineurin signaling. For instance, a study assessing the physiological roles of estrogen-related receptor gamma (ERR*γ*), an orphan nuclear receptor highly expressed in the nervous system during embryogenesis and over lifespan, revealed its involvement in the regulation of dopaminergic neuronal phenotype. Such effect was mediated by GSK-3*β*-NFAT interactions, independently of Ca^2+^/calcineurin signaling [139]. Similar, NGF facilitated NFAT-mediated gene expression induced by mild depolarization in dorsal root ganglion sensory neurons without changes in PLC activity-dependent events, including Ca^2+^/calcineurin signaling. Instead, NGF effects were induced by phosphoinositide 3-kinase (PI3K)/Akt signaling triggered by TrkA receptor activation, which abrogated GSK-3*β* activity [77].

Furthermore, calcineurin is universally involved in vesicle endocytosis [12], and alterations in its endocytic activity may participate in the pathogenic processes of various psychiatric diseases. For instance, alterations in presynaptic functions of the *γ* isoform of the calcineurin catalytic subunit, such as synaptic vesicle cycling, have been suggested to contribute to schizophrenia, where variations in calcineurin A*γ* gene* PPP3CC* are common in neurons [140]. Deregulation of intracellular Ca^2+^ also associates with the disruption of fast axonal transport (FAT) in the pathogenesis of Alzheimer's disease (AD) [141–144].

## 4. Nervous System Function

**Figure 1:**
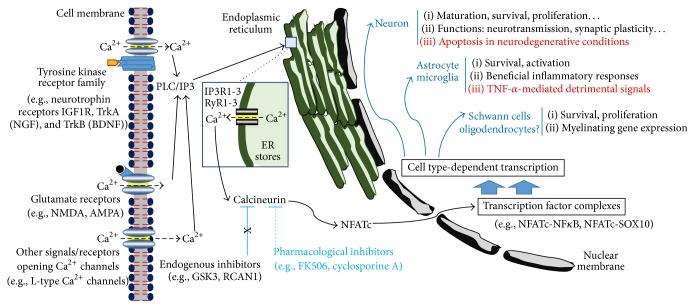
Calcineurin/NFAT signaling pathway. Calcineurin/nuclear factor of activated T-lymphocytes (NFAT) signaling activation in the nervous system is mainly induced by neurotrophins via their tyrosine kinase receptors, glutamate receptors, and nonligand-dependent receptors, such as voltage-gated Ca^2+^ channels in hippocampal neurons. The genes transcribed and the effects of these signaling pathways are cell-type dependent. Abbreviations: AMPA, *α*-amino-3-hydroxy-5-methyl-4-isoxazolepropionic acid; BDNF, brain-derived neurotrophic factor; GSK3, glycogen synthase kinase 3; IGF1R, insulin-like growth factor 1 receptor; IP_3_R, inositol 1,4,5-trisphosphate receptor; NF-*κ*B, nuclear factor kappa B; NFATc, Ca^2+^-regulated NFATs; NGF, neurotrophin nerve growth factor; NMDA, N-methyl-D-aspartic acid; RCAN1, regulator of calcineurin 1; RyR; ryanodine receptor; TrkA, tropomyosin receptor kinase type 1; TrkB, tropomyosin receptor kinase type 2.

Calcineurin/NFAT has critical roles in neuronal and glial cell activity and survival, as well as resulting events, fundamental for nervous system function like neurotransmission and synaptic plasticity, and myelination as summarized in Figure 1.

### 4.1. Synaptic Plasticity and Neurotransmission

#### 4.1.1. Synaptic Connectivity and Plasticity

Calcineurin/NFAT signaling and other pathways activated by T-type Ca^2+^ channel activation play critical roles in the shaping of synaptic connectivity of thalamocortical and nucleus reticularis thalami GABAergic neurons mediated by slow wave sleep [145], a process pivotal for the consolidation of recently acquired memories and for the restoration of synaptic homeostasis. This mechanism may be evolutionary conserved [54, 146, 147]. PLC/calcineurin signaling has been reported to regulate the trafficking of GABA A receptor in layer 3 pyramidal cells of murine barrel cortex [136]. Furthermore, the inhibition of group I metabotropic glutamate receptors (mGluRs), IP3Rs, or calcineurin in CA1 neurons resulted in the blockade of the heterosynaptic shrinkage [85, 87] that drives circuit remodeling during activity-dependent refinement of the developing nervous system and during experience-dependent plasticity in the hippocampus. This inhibition has been reported to negatively affect long-term potentiation (LTP) [87].

Experimental evidence also suggests that calcineurin is a regulator of synaptic plasticity. For instance, axon initial segment rapid shortening was partly mediated by calcineurin-dependent mechanisms, including phosphorylation of voltage-gated Na^+^ channels, in dentate granule cells [148]. Rapid modulation of the axon initial segment, the site of action potential initiation, is the major plasticity mechanisms used by neurons to control their excitability time from seconds to days. In another study, decreases in resting Ca^2+^ levels associated with prolonged blockade of synaptic activity resulted in the synthesis of retinoic acid, which triggered the related homeostatic synaptic plasticity, via a calcineurin-dependent mechanism in neurons [149]. Moreover, spinogenesis enhancement in hippocampal neurons by the steroid hormones dihydrotestosterone and testosterone was blocked by individual antagonism of PKC, PKA, calcineurin, LIM kinase (LIMK), or the MAPKs Erk and p38 [150]. Similar, estradiol-mediated rapid modulation of synaptic plasticity, an essential process for synaptic regulation, was abrogated by individual targeting of PI3K, PKC, PKA, calcineurin, CaM kinase II (CaMKII), LIMK, Erk, or p38, in hippocampal neurons [151].

#### 4.1.2. Learning and Neurotransmission

Evidence of calcineurin/NFAT involvement in learning and memory processes was provided by experimental models of neurodegenerative disorders. A study in PS1-M146V knock-in FAD mice showed that decreased calcineurin activity is a common phenomenon in aging-related memory decline and may account for memory defects in AD, together with mutations in the gene encoding for GSK-3*β* substrate presenilin 1 (PS1) [152], which are critical for amyloid-*β* (A*β*) generation. In a study using a spinocerebellar ataxia type 3 (SCA3) transgenic mouse model, typical impairments of motor learning and cerebellar motor coordination resulted from altered long-term depression (LTD) of glutamatergic transmission in parallel fiber-Purkinje neurons [153]. Such alteration resulted mainly from transcriptional downregulation of PLC *β*4, IP3R1, and calcineurin B, suggesting that PLC/IP_3_-Rs/calcineurin signaling is required for cerebellar LTD induction, thus motor learning and coordination.

Calcineurin/NFAT signaling is also involved in neuronal excitability and neurotransmission. Under physiological conditions, AKAP79/150-mediated calcineurin/NFAT signaling may prevent neuronal hyperexcitability in hippocampal neurons by increasing the transcriptional expression of key regulators of neuronal excitability like M-type K^+^ channels [154]. PKC/calcineurin/NFAT signaling contributes to the maintenance of cyclic nucleotide-gated (HCN) channels in the hyperpolarized status critical for their mediation of neuronal excitability decrease in the distal dendrites of hippocampal CA1 pyramidal neurons [155], and membrane-derived bioactive phospholipid lysophosphatidic acid type 1 (LPA1) triggers RhoA/Rho kinase (ROCK)/calcineurin signaling to induce the internalization of the GABAA*γ*2 subunit at inhibitory synapses [156].

### 4.2. Calcineurin-PKA Interactions

The interplay between calcineurin and PKA signaling plays a critical role in the negative-feedback mechanism driving homeostatic synaptic plasticity [157], that is, accounting for the compensation of excessive inhibition or excitation of neuronal activity. Although calcineurin/NFAT signaling mainly regulates axon terminal remodeling, while PKA/CREB signaling controls synaptic vesicle accumulation [158], many lines of evidence suggest mutual inhibitory interactions between the activities of these signaling pathways. Examples include the activity of these signaling molecules when anchored to AKAP79/150. PKA kinase activity triggered by anchoring to AKAP79/150 resulted in the enhancement of Ca^2+^-dependent inactivation of L-type Ca^2+^ channels, while the activation of the phosphatase activity of AKAP79/150-anchored calcineurin reversed such PKA action, reducing Ca^2+^-dependent inactivation [96, 159]. The basal activity of AKAP79/150-anchored PKA maintained L-type Ca^2+^ channel-calcineurin/NFAT signaling functional coupling by preserving the phosphorylation of these channels, contrary to anchored calcineurin [159, 160]. Interestingly, such AKAP79/150 activity mediated the modulation of roundabout axonal guidance receptors Robo2/3 and ligands Slit2/3 in brain regions involved in reward, learning, and memory processes like islands of Calleja and the hippocampus [97].

Furthermore, PKA can induce NFAT nuclear export [14, 54]. PKA activation by forskolin-stimulated cAMP increased the stability and half-life of RCAN1 protein, enhancing its inhibitory effects on calcineurin [161]. In a recent study in pilocarpine-induced status epilepticus, a murine model of temporal lobe epilepsy (TLE), the nuclear translocation of CREB-regulated transcription coactivator 1 (CRTC1), which is a key regulator of CREB activity, was regulated by calcineurin activity in hippocampal neurons [24].

Opposite effects of calcineurin and PKA were also reported as key events in (i) the dynamic fission-fusion events that determine the shape and function of mitochondria [162, 163], thus cell survival; (ii) neuronal output stabilization induced by tonic dopamine via type 1 dopaminergic receptors [164]; and (iii) the phosphorylation/dephosphorylation of serine 897 in the NR1 subunit of the NMDA receptor (pNR1), whose increases in phosphorylation were reported in acute morphine withdrawal [30].

### 4.3. Myelination

Available data suggest that calcineurin/NFAT pathway participates in signaling cascades pivotal for Schwann cell myelination. Early studies reported that (i) murine Schwann cells express all Ca^2+^-dependent NFAT isoforms; (ii) the promoter and upstream enhancer elements of the myelinating factor Krox-20 contain NFAT binding sites; and (iii) NFATs recruited by Ca^2+^-dependent signaling can make transcriptionally active complexes with Krox-20 [9, 93, 165]. Reporter assays showed that Krox-20 is NFAT target gene and that calcineurin is upregulated in Schwann cells expressing Krox-20 [8, 166]. Moreover, we reported using rat Schwann cell cultures and an* in vitro* model of myelination that promyelinating actions of calcineurin/NFAT signaling, including increases in the expression of the myelinating genes* Krox-20*,* Periaxin,* and* P0*, require cAMP elevation [9]. Notably, in the absence of cAMP elevation, increase in cytosolic Ca^2+^ failed to induce Krox-20 expression. Furthermore, cyclosporine A and FK506 abrogated Krox-20 expression. Comparable observations were reported more recently by other authors [165, 167].

Experimental evidence also suggests that the activation of calcineurin/NFAT required for Schwann cell myelination occurs partly through neuregulin 1 (NRG1) stimulation [8, 168–170]. A study in mice lacking calcineurin B in cells of the neural crest lineage showed that calcineurin/NFAT signaling is required for NRG1-mediated Schwann cell myelination [170]. In this model NFAT activation failed, Krox-20 levels in Schwann cell were decreased, and radial sorting and myelination were markedly delayed [8, 170]. NRG1 addition to neuron-Schwann cell cocultures promoted the activation of NFAT isoforms and cooperative transcriptional activities of NFATc4 and SOX10 required for Krox-20 upregulation [168, 169]. In addition, NRG1/calcineurin/NFAT signaling upregulates myelinating genes in Schwann cells [8, 168, 171].

Unexpectedly, FK506 stimulated Schwann cell proliferation and promoted the survival of oligodendrocyte in murine models of traumatic spinal cord injury [172–174], suggesting that this signaling pathway is involved in the maintenance, thus activity, of myelinating cells in both peripheral and central nervous systems. Moreover, NFAT1 hyperactivation decreased experimental autoimmune encephalomyelitis induced by myelin oligodendrocyte glycoprotein (MOG), a key regulator of CNS myelination [175, 176].

## 5. Controversial Roles in Nervous System Diseases

### 5.1. Neurodegenerative Diseases

#### 5.1.1. Pathogenic Roles

Increased calcineurin activity was reported in both aging and AD models [27, 177, 178]. For instance, calcineurin/NFAT signaling may mediate the aberrant activity of deregulated plasma membrane Ca^2+^ pumps (PMCAs), a suggested link between brain aging and the onset of neurodegenerative diseases [27, 179, 180]. The strongest AD genetic risk factor, the apoE4 allele, encodes for apolipoprotein E4 that has poor inhibitory abilities on calcineurin activity, unlike the neuroprotective apolipoproteins E2 and E3 [181–183]. Apolipoprotein E4 also drives CNS functional alterations associated with normal aging such as disturbed sleep [28, 29].

Overactivated calcineurin/NFAT signaling may contribute to synaptic plasticity affection in pathological conditions. Notably, in a postmortem study in human hippocampi, high nuclear levels of NFATs observed at the early stage of AD increased with cognitive decline severity [177]. Additionally, short exposure to A*β* oligomers resulted in calcineurin activation with transient changes in postsynaptic proteins and morphological in spines, while longer exposure resulted in NFAT activation and marked spine loss in primary cortical neurons of wild-type mice [184]. A*β*-treatment of murine hippocampal neurons also resulted in Ca^2+^ signaling-dependent defects in BDNF transport first in dendrites and then in axons [40]. Studies in mouse and rat models of severe childhood epilepsy revealed that calcineurin/NFAT signaling mediates seizure-induced dendrite growth suppression in pyramidal neurons and thus the resulting learning and memory impairment associated with this intractable condition [185].

NFAT signaling alterations in neurodegenerative diseases are selective. A report by Abdul and colleagues strongly suggested that such selective alteration may play a key role in A*β*-induced neurodegeneration [186]. These authors observed increases in calcineurin A activity and more marked shifts of NFATc2 and NFATc4 to nuclear compartments in human hippocampus with increased dementia severity, while even in rapid-autopsy postmortem human brain tissue NFATc1 was unchanged. NFATc2 was more active in AD patients with mild cognitive impairment, contrary to NFATc4 whose expression was mostly associated with severe dementia. Still in the same study, changes in calcineurin/NFAT4 were directly correlated to soluble A*β* levels in postmortem hippocampus, while oligomeric A*β* strongly stimulated NFAT activation in primary rat astrocyte cultures. In another study, NFATc4 levels were significantly increased in brains of APP/PS1 transgenic mice (AD model) and NFATc4 overexpression increased A*β* production in human myeloid leukemia SAS-1 cells [36], suggesting a role for NFATc4 in amyloidogenesis. Mechanisms proposed for NFAT-mediated amyloidogenesis in human and murine astrocytes include increases in the expression of the gene encoding for TMP21, a p24 cargo protein involved in A*β* and A*β* precursor protein (APP) trafficking [187–190].

#### 5.1.2. Beneficial Effects

The role of calcineurin in aberrant *α*-synuclein-mediated midbrain dopaminergic neuron toxicity, a hallmark of Parkinson's disease (PD), is controversial. In a study addressing the underlying intracellular mechanisms driving *α*-synuclein-mediated neurodegeneration, transgenic expression of PD *α*-synuclein A53T missense mutation promoted calcineurin/NFAT signaling, suggesting that this signaling pathway may contribute to the neurotoxic effects of aberrant *α*-synuclein [191]. Surprisingly, in a study using cells from various models (ranging from yeast to neurons), although aberrant *α*-synuclein also seemed to induce cellular toxicity via overactivation of Ca^2+^-dependent signaling pathways, calcineurin inhibition with FK506 also resulted in toxicity [192], suggesting that calcineurin may mediate both beneficial and toxic effects under stimulation by aberrant *α*-synuclein. Characterizing the precise downstream targets mediating calcineurin beneficial or toxic effects may provide more insights of the novel therapeutic targets for synucleinopathies.

Calcineurin also mediates some beneficial and toxic effects of AMPA and NMDA receptors, which are postsynaptic site-located glutamate-gated ion channels critical for synaptic plasticity. Calcineurin translocation to synapses and increases in its activity mediated by NMDA receptor trafficking were reported as critical components of mechanisms driving rapid compression-induced dendritic spine plasticity in cortical pyramidal neurons, that is, the rapid trimming of dendritic spines occurring about 12 hours after mechanical compression [38]. A study using soluble A*β* treated in cultured rat hippocampal neurons and cultured hippocampal neurons from APPSwe AD-transgenic mice suggested that calcineurin signaling mediates AD-like synaptic dysfunction induced by tau protein partly via AMPA receptor downregulation [193]. In that study, soluble A*β* oligomer-induced deficits in AMPA receptor trafficking were mediated by tau phosphorylation and mislocalization to dendritic spines. FK506 abrogated all these alterations. Concomitant tau hyperphosphorylation and calcineurin overactivation were also reported in mouse models of Huntington's disease [41]. On the other hand, IL-6/Janus kinase (JAK) signaling induced neuroprotective anti-NMDA activities in cultured cerebellar granule neurons via calcineurin-dependent inhibition of activities of NMDA receptor subunits NR2B and NR2C and concomitant inhibitions of NMDA-induced L-type voltage-gated Ca^2+^ channel activity and intracellular Ca^2+^ store release [194].

Moreover, in AD pathogenesis, insulin-like growth factor 1 (IGF-1) that acts as a regulator of tau phosphorylation is silenced in activated astrocytes by A*β*/calcineurin-induced release of IGF-1-binding protein 3 (IGFBP-3); but intriguingly, A*β* directly induces increases in tau phosphorylation, and resulting neuronal death, via a mechanism involving the silencing of NFAT export kinase GSK-3*β* [195], suggesting opposite roles for calcineurin in AD pathogenesis. IGF-1 also protected motor neurons in SOD1 transgenic mice, a widely used model of amyotrophic lateral sclerosis, via a calcineurin-dependent mechanism [196]. Although it is now widely accepted that therapeutic benefits of IGF-1 treatment in neurodegenerative conditions may emerge partly from calcineurin-dependent inhibition of glial inflammatory reaction mediated by preventing TNF-*α*-induced nuclear translocation of NF-*κ*B [197–199], it is also clear that the expression of TNF-*α*, resulting in detrimental neuroinflammation and functional alterations in neurons, is mediated by astrocytic and microglial calcineurin/NFAT signaling [200–202]. Studies in transgenic mice and* in vitro* models of neuroinflammatory diseases provided mechanistic insights into the context of these opposite roles. These studies showed that TNF-*α* activate calcineurin/NFAT/NF-*κ*B canonical inflammatory pathway in quiescent astrocytes, while in activated astrocytes, IGF-1 released locally recruited calcineurin signaling to inhibit NF-*κ*B-NFAT transcriptional activity through activation of the purinergic receptor P2Y6 [203, 204], suggesting that the activation status of the cell is an important determinant of calcineurin/NFAT activity, that is, downstream targets.

#### 5.1.3. Pharmacological Inhibition and Endogenous Regulation

The pharmacological inhibition of calcineurin/NFAT signaling improved animal condition in a number of studies in neurodegenerative diseases and models. Examples include AD mouse models where pharmacological inhibition of this signaling pathway decreased A*β* plaques, reduced glial activation, alleviated both A*β* synaptotoxicity and neurotoxicity, and improved synaptic function [25, 205–207], suggesting a therapeutic potential for calcineurin inhibitors in AD. Reports by Kim and colleagues from studies performed in presenilin 1-mutant model of AD provided some mechanistic insights into the cognitive decline improvement resulting from reducing calcineurin activation in affected brains [25, 208]. These authors observed that the inhibition of abnormally increased calcineurin activity characteristic of the disease resulted in the stabilization of the phosphorylation of GluA1, a subunit of Ca^2+^-permeable AMPA receptors, and promoted synaptic trafficking of Ca^2+^-permeable AMPA receptors, as well as the resulting improvement in animal cognition [25]. Such improvement resulted at least partly from the restoration of Ca^2+^-permeable AMPA receptor-mediated hippocampal LTP [208]. Decrease in calcineurin complexes with transmembrane AMPA receptor regulatory proteins (TARPs) like *γ*-8, which can stop the trafficking of both AMPA and to a lesser extent NMDA receptors [209–211], may also participate in this process. Furthermore, biometal mediated neurite elongation and neuritogenesis in neuron cultures via calcineurin silencing [111] and calcineurin/NFAT signaling induced a reduction in NGF expression and neurite outgrowth in rat neonatal ventricular cardiomyocytes and cultured sympathetic neurons [212, 213].

Not surprisingly considering the aforementioned observations in studies assessing the roles of calcineurin in neurodegenerative disorders (Section 5.1.2), endogenous regulators like plasma membrane calcium ATPase (PMCA) and RCAN1 mediate opposite effects in inflammatory processes. RCAN1 overexpression was reported to be pivotal in the prevention of sepsis and LPS-induced lethality [214] and in the protection against brain ischemia/reperfusion injury in murine models [215]. However, interactions of PMCA and vascular endothelial growth factor (VEGF), which dampened calcineurin/NFAT signaling, also induced the overexpression of both the counter-inflammatory factor RCAN1.4 and the proinflammatory factor COX-2 in activated murine endothelia [214, 216, 217]. Additionally, RCAN1 overexpression increased the susceptibility to oxidative stress in primary neurons [218] and exacerbated Ca^2+^ overloading-induced neuronal apoptosis [219], suggesting that the overexpression of calcineurin regulator RCAN1 may link Ca^2+^ overloading and oxidative stress in neurodegenerative disorders (Figure 1). Such detrimental effects of RCAN1 were mediated by RCAN1.4, and not by the other isoforms detected in human brain RCAN1.1 [91, 219], probably via PI3K/Akt/mTOR signaling [220, 221]. Such mechanistic insight was provided by a system study based on combinations of single-cell experimentation and* in silico* simulations where RCAN1 effect on inflammation mediated by calcineurin/NFAT appeared to change according to cellular levels, from inhibitory activity at low levels to facilitative activity at high levels [221]. Notably, in that study RCAN1 facilitative activity was switched on by nuclear export of GSK-3*β*, indicating that targeting the factors involved in this inhibitory mechanism of GSK-3*β*-mediated NFAT nuclear export may have a therapeutic potential in neurodegenerative diseases.

Considering that RCAN1 overexpression is a hallmark of Down syndrome [219, 222], it can be hypothesized that this event also contributes to the pathogenesis of AD-like neuropathology typically observed in Down syndrome patients after their middle age [223, 224]. In addition, the activity of pituitary adenylate cyclase-activating peptide (PACAP), a neurotrophic peptide involved in nervous system development, learning, and memory, was significantly disturbed by changes in RCAN1 expression [225]. RCAN1 overexpression impaired neurotrophic support of sympathetic neurons by inhibiting TrkA endocytosis, resulting in NGF signaling silencing and associated neurodevelopmental deficits [92]. Furthermore, overexpression of RCAN1 or dual-specificity tyrosine-(Y)-phosphorylation regulated kinase 1A (DYRK1A), another Down syndrome-associated protein, negatively regulated NFAT-dependent transcriptional activity and decreased NGF-mediated upregulation of PAI-1 levels [131], a key synaptogenic mechanism. Intriguingly, deficiency of RCAN1.1, but not RCAN1.4, affected radial migration of rat cortical neurons and caused periventricular heterotopia [226], suggesting that this RCAN1.1 isoform may mediate positive RCAN1 effects in developing cortex. Future studies using selective activations and inhibitions of RCAN1 isoforms may reveal the mechanisms accounting for isoform-specific effects in the developing cortex.

### 5.2. Neurodegenerative Conditions and Other Nervous System Diseases

#### 5.2.1. Neurodegenerative Conditions

Alterations in the autophagy of mitochondria, the process that normally triggers damaged organelle elimination, are common in neurodegenerative diseases and conditions [227–230]. In a study using axotomized precerebellar neurons, a model of focal cerebellar lesion-induced remote degeneration, rapamycin-mediated autophagy, resulted in an aberrant mitochondrial fission partly caused by increased calcineurin activity [227]. The activity of a calcineurin docking motif present in the mitochondrial fission mechanoenzyme dynamin-related protein 1 (Drp1) contributed to mitochondrial fragmentation and ischemic neuronal injury in neuronal and nonneuronal cells [163]. Additionally, calcineurin inhibitors mitigated mitochondrial fragmentation in ferric ammonium citrate-exposed HT-22 hippocampal neurons, a model of iron overload and neurodegeneration [231].

Further evidence for calcineurin involvement in ischemic injury includes reports suggesting that abnormal increases in the activity of this phosphatase, mediated by disturbances in axonal Ca^2+^ homeostasis, may play a key role in secondary damage of neurons and capillary vessels observed during acute phase of diffuse axonal injury [173, 232]. Calcineurin signaling also mediated the activation of the cytoskeletal actin severing protein cofilin and the resulting neuronal death in oxygen-glucose deprivation/reperfusion and chemical induced oxidative stress, to* in vitro* models of ischemia [34]. Moreover, cyclosporine A prevented the apoptosis of astrocytes exposed to simulated ischemia* in vitro* via a calcineurin and Erk1/2-dependent mechanism [233] and through the inhibition of cytosolic phospholipase A2- (PLA2-) mediated release of arachidonic acid [234].

Selective calcineurin signaling in neurons and astrocytes is a key player in neurodegenerative conditions. An early study addressing neuronal apoptosis induced by the abused psychostimulant methamphetamine revealed pivotal roles for calcineurin activation and resulting Fas ligand upregulation mediated by nuclear translocations of NFATc3 and NFATc4 in rats [235]. The potent hepatotoxin microcystin-LR (MCLR) mediated an upregulation of calcineurin and NFATc3 levels in rat hippocampal neurons that resulted in marked increases in apoptotic and necrotic cell death [32]. MCLR effect was prevented by FK506 treatment. In addition, NFATc4 mediated light-induced retinal ganglion cell apoptosis by upregulating Fas ligand (FasL) expression on retinal neurons [236], and the overactivation of calcineurin/NFATc3 signaling induced the typical neuronal toxicity and functional alterations observed in murine developing hippocampal neurons following the inhalation of anesthetic isoflurane, including cognitive impairment [237]. A recent study in this model of postoperative cognitive dysfunction revealed that abnormal calcineurin/NFAT signaling associated with isoflurane exposure may mediate its detrimental effects by promoting the degradation of the survival molecule signal transducer and activator of transcription 3 (STAT3) [238]. Moreover, calcineurin/NFATc3 signaling in activated astrocytes played a key role in the induction of alterations in synaptic remodeling and homeostasis observed in the hippocampus in controlled cortical impact injury in rats [39, 128, 239]. As expected, calcineurin inhibition restored synaptic function and plasticity in the latter murine model of traumatic brain injury and in murine models of traumatic spinal cord injury [172–174] partly by abrogating astrocyte activation and reactive gliosis, which are pivotal events in neuroinflammation-mediated neuronal loss. Notably, calcineurin/NFAT signaling is critical for astrocyte activation [205, 240]. In another study in ischemic striatum and cortex and in cultured astrocytes where FK506 also induced neuroprotective effects, the calcineurin inhibitor prevented astrocyte apoptosis mediated by glutamate signaling [241].

Altogether, these observations suggest a role for calcineurin/NFAT signaling in astrocyte and neuronal losses observed in nervous system injury. Unexpectedly, sublethal ischemia increased neuronal resistance to excitotoxicity via calcineurin-dependent mechanisms including cyclin E1 protein increased expression and declustering of the delayed rectifying K^+^ channel Kv2.1 at highly phosphorylated somatodendritic clusters [242]. In addition, preconditioning of neurons with biometal ions (Cu^2+^, Zn^2+^) protected these cells against NMDA receptor-induced excitotoxicity, through metal chaperone PBT2-induced calpain cleavage of calcineurin [111, 112]. Thus, calcineurin/NFAT pathway participates in the interplay between proinflammatory and counter-inflammatory signals in the nervous system, further suggesting that unraveling the downstream targets accounting for beneficial and neurotoxic effects of this signaling pathway may have a therapeutic potential in neurodegenerative conditions.

#### 5.2.2. Psychotic Disorders

Early genetic studies showed that polymorphisms of the genes coding for either the catalytic or regulatory subunit of calcineurin isoenzymes are strongly associated with the risk for developing schizophrenia and other psychotic disorders whose pathological features include disturbances in Ca^2+^ signaling [46, 243–246]. A more recent genome-wide weighted coexpression network analysis on neural progenitors and neurons from individuals with Timothy syndrome, an autism spectrum disorder resulting from mutations in the gene encoding L-type CaV1.2 Ca^2+^ channels, suggested that the disease may be caused by disturbances in transcriptional activities of Ca^2+^-dependent signaling molecules like FOX proteins, MEF2, CREB, and NFATs [247].

Furthermore, GABA A receptor activation promoted a decrease in anxiety indicators and hippocampal neurogenesis via the calcineurin/NFAT4 signaling in mice, suggesting that pharmacological targeting of this signaling pathway may improve emotional disorders [248]. Similar evidence from pharmacological and postmortem studies suggests that treatment with antipsychotics aimed at ameliorating some of the symptoms of the CNS disorders leads to alterations of the calcineurin expression pattern in the human brain [249–253]. Decreased calcineurin levels in the nucleus accumbens were reported in opioid withdrawal, a dysphoric state associated with complications in patient pain and increased risk of drug abuse and addiction [30], suggesting a role for calcineurin in long-lasting behaviors associated with reward. Furthermore, in a patch-clamp electrophysiology and fast-scan cyclic voltammetry study in mouse brain slices, the endogenous modulatory peptide neurotensin induced a long-term prevention of pathogenic increases in presynaptic dopamine release, characteristic of schizophrenia and other severe mesencephalic pathologies, by increasing inhibitory D2 dopamine autoreceptor function via a calcineurin-dependent mechanism [35].

#### 5.2.3. Epilepsy

Many lines of evidence also support calcineurin involvement in nervous system diseases. Notably, various reports have suggested that calcineurin is likely to mediate physiological and pathological activities of GABA receptors. For instance, complex interactions between PKC and calcineurin may play a key role in GABA B autoreceptor-mediated functional regulation of nicotinic acetylcholine receptors (nAChRs), whose activation triggers the release of neurotransmitters from presynaptic nerve terminals, in mouse striatal GABAergic nerve terminals [254]. Somatic modulation of GABA A receptor-mediated fast inhibitory signaling in epileptiform activity was induced by calcineurin signaling in low-magnesium model of seizure in rat hippocampal neurons [31], suggesting a role of calcineurin in benzodiazepine resistance and the potential of its pharmacological targeting in status epilepticus.

Certainly, calcineurin/NFAT signaling involvement was also shown in pathogenic processes of other models of status epilepticus, including intracerebral injection of kainic acid [255], bicuculline [256], and pilocarpine [33]. Notably, calcineurin inhibitor ascomycin mediated anticonvulsant and neuroprotective effects, in different epilepsy models, including picrotoxin and latrunculin A models [257–259].

### 5.3. Emerging Challenge: Better Models?

A growing number of studies are raising concerns about mechanistic reports of Ca^2+^-dependent and other signaling pathways from currently used models of neurodegenerative diseases, in particular transgenic animals and cell lines. A study addressing the suitability of rat striatal primordia-derived ST14A cell line for the study of voltage-gated Ca^2+^ channel of striatal medium spiny neurons called for serious caution on the assumption of the presence of complete signaling cascades of G-protein coupled receptors in cell lines [260]. Notably, ST14A cells were reported to lack PLC-*β*1, a major effector of G-proteins for Ca^2+^ release from intracellular stores [59, 82], whose roles include (i) the regulation of forward locomotion in wild-type mice, among other dopamine receptor functions [261]; (ii) the mediation of Ca^2+^ flux required for mammalian sperm acrosome reaction [262]; and (iii) the mediation of the positive regulation of osteoblast differentiation [263]. In addition, most observations from studies in preclinical models of neurodegenerative diseases based on exogenous neurotoxins are not confirmed by clinical studies [264–266]. Similar observations emerge from studies in transgenic animals, where complex functional adaptations from gene knock-in or knock-out may limit the translational importance of findings [267–269]. In the case of neurodegenerative conditions, at least some of the controversy regarding changes in calcineurin/NFAT activity and/or expression appears to be due to the methodologies employed to measure activities and expression levels. Calcineurin, in particular, is highly sensitive to proteolysis during injury and neurodegeneration. However, most commercial antibodies to calcineurin only detect full-length calcineurin and miss the detection of high activity proteolytic fragments. Also, many studies have tended to measure calcineurin activity in whole brain tissue homogenates using commercially available phosphatase assays. While these kinds of assays are very good for kinetic analyses, they are very poor at assessing endogenous calcineurin activity toward endogenous substrates. Certainly, methodological challenges may have affected the quality of data generated by different groups. Although it appears that better models are needed, we also propose that better characterization of intracellular signaling in currently available and future experimental models may improve the translational importance of the findings.

## 6. Concluding Remarks

Calcineurin/NFAT pathway is pivotal during nervous system development and in various functions of mature central and peripheral nervous system. Notably, this signaling pathway is involved in myelination, corticogenesis, synaptogenesis, neuritogenesis, endosome trafficking, homeostatic synaptic plasticity, learning, and memory. Experimental evidence also shows that alterations in the activity of calcineurin/NFAT pathway and in activities of its endogenous regulators in the nervous system microvascular endothelial cells, astrocytes, microglia, Schwann cells, oligodendrocytes, and neurons participate in the pathogenesis of neurodegenerative diseases and conditions, but also psychotic disorders. Studies in transgenic animals and in cell lines also suggested that neurodegeneration-associated detrimental changes in calcineurin/NFAT signaling are NFAT isoform-selective as changes in NFATc3 and NFATc4, but not NFATc1 or NFATc2, are usually common. In addition pharmacological inhibition mitigated neuronal and astrocyte loss and improved cognitive functions in many models. However, other studies reported beneficial roles of calcineurin/NFAT in neurodegenerative diseases and conditions, in particular those reporting neurotoxic effects of pharmacological inhibition and increased endogenous regulation. These studies suggested that calcineurin can mediate both neuroprotective and neurodegenerative signals according to poorly understood determinant factors, which included the activation status of astrocytes in the central nervous system. Future studies should be devised to characterize better the factors determining the outcome of calcineurin/NFAT signaling in neurodegenerative diseases and conditions, as well as the downstream targets mediating the beneficial and detrimental effects of this signaling pathway, considering the implications for therapy.
